# Heme disposal inside heart transplants: a trigger for rejection?

**DOI:** 10.1172/JCI201815

**Published:** 2026-02-02

**Authors:** Fuyi Liao, Andrew E. Gelman

**Affiliations:** Department of Surgery, Washington University School of Medicine, St. Louis, Missouri, USA.

## Abstract

Cardiac allograft vasculopathy (CAV) is a fibroproliferative form of transplant rejection with limited treatment options other than retransplantation. In this issue, See and colleagues examined human explanted allografts with CAV. They found that a high proportion of intragraft plasma cells produce antibodies that recognize the heme catabolic end product, bilirubin. Clonotypic profiling revealed that bilirubin-reactive antibody-producing plasma cells develop from graft-infiltrating innate-like B cells, a subset often characterized by their rapid production of polyreactive natural antibodies as an early defense against infection. CAV but not nonrejecting graft tissue contained bilirubin deposits along with macrophages that expressed genes involved in heme catabolism. These findings raise the intriguing possibility that graft-derived bilirubin-specific antibodies target local heme catabolism to promote CAV.

## Profiling intragraft innate-like B cell differentiation during rejection

Cardiac allograft vasculopathy (CAV) is a major cause of heart transplant rejection and is associated with dense immune infiltration in the coronary arteries. It is well established that rejecting cardiac allografts have intragraft class-switched B cells and plasma cells (PCs), with early work indicating that these cells participate in donor-specific recognition of human leukocyte antigen (HLA) (1). However, with the advent of single-cell gene profiling techniques, accumulating evidence suggests this may not be the case. Emerging observations indicate that chronically rejected human lung, kidney, and heart transplants contain activated B cell subsets with transcriptional profiles more akin to those of mouse B-1 B cells and human innate-like B cells than to those of conventional B cells (2–4). In contrast with conventional B cells, that are better suited to making high-affinity donor-specific HLA antibodies, innate-like B cells are best characterized by their production of natural antibodies, which are critical for rapid responses to infections. Whereas conventional B cells recirculate throughout the body, innate-like B cells are primarily localized to specific tissues or body cavities. Innate-like B cells are also distinguished by their polyreactive antigen specificity, which expands their clonal capacity through the recognition of particular chemical adducts attached to what can be structurally dissimilar macromolecules (5).

In the transplant setting, recent work has indicated that intragraft innate-like B cells, in part, target organ-specific antigens. Antibodies expressed from isotype-switched immunoglobulin sequences in innate-like B cells from rejecting human kidney allografts showed greater affinity for renal-specific antigens than for donor HLA, even in recipients with circulating donor-specific antibodies (3). However, whether there is an expansion of innate-like B cells with organ-antigen specificity in other rejecting organs remains unclear. Additionally, it remains to be determined the degree to which activated intragraft innate-like B cells and PCs are clonally related.

In this issue of *JCI*, See et al. analyzed antigen specificities of B cells and their clonal relatedness to PCs in explanted human heart transplants with and without CAV, as well as in the peripheral blood (6). Using single-cell RNA profiling, they first compared peripheral blood B cells and PC populations to their intragraft counterparts. Unlike B cells and PCs in control heart tissue, CAV intragraft B cells and PCs exhibited transcript accumulation characteristic of innate-like B cells, including *AHNAK*, a gene reported to be preferentially expressed in mouse B-1 B cells and human innate-like B cells from rejecting kidney allografts. Additionally, gene expression enrichment analysis identified pathways associated with B cell activation and antibody secretion, suggesting local clonal expansion and differentiation into PCs. To explore this finding further, the group conducted clonotypic profiling on CAV specimens and PBMCs using a combination of 5′ single cell V(D)J’ scRNA analysis and DNA sequencing on rearranged immunoglobulin γ heavy chain (IGH) variable region genes. Cells were considered clonally related if they had the same IGH variable and joining segment usage and a greater than 85% sequence similarity in complementary-determining region 3. In line with earlier work by these investigators, clonal relatedness was nearly absent between CAV B cells and plasma cells compared with the same respective cells in peripheral blood (4). However, almost a third of intragraft PCs and B cells were clonally related, supporting the notion that intragraft innate-like B cells differentiate locally into PCs during CAV pathogenesis.

## Bilirubin deposits as local antigenic targets in CAV

Previous work from this group has shown that B cells in CAV lesions exhibit polyreactive antigen profiles characteristic of innate-like B cells (4). However, the specificities of antibodies encoded by intragraft PCs remain largely unexplored. To profile antigen specificities in the present study, the group generated monoclonal antibodies (mAbs) from PCs that showed evidence of expansion and isotype switching by selecting cells with paired immunoglobulin γ heavy and light chains that were rearranged and detected multiple times. Then the investigators drew upon their previous work (4), which showed that innate-like B cell polyreactivity results from recognition of specific chemical adducts covalently linked to various macromolecules. Here, using their unique high-dimensional ELISA platform, they tested 37 PC-generated mAbs for reactivity against a 93-adduct panel. More than half of these mAbs bound the end-product of heme catabolism, bilirubin. In contrast, only 1 of 96 mAbs derived from circulating memory B cells of recipients with CAV detected bilirubin, indicating that bilirubin is a primary antigen target of intragraft antibody production during CAV pathogenesis.

Interestingly, there are no prior reports of bilirubin accumulation in rejecting hearts, although deposits have been observed in atherosclerotic lesions in experimental models (7). Using commercially available bilirubin-specific antibodies, See and colleagues detected bilirubin deposits in CAV specimens, with the bulk of reactivity localized to immune cell infiltrates and to smooth muscle cells lining the coronary arteries. Bilirubin was also found in biopsies from a patient who later developed CAV, suggesting it could also be an early indicator of the disease. In contrast, bilirubin deposits were not found in cardiac allografts without CAV or in healthy hearts from patients who did not die of cardiac disease, further supporting the association between bilirubin and CAV development. Finally, intragraft PC-derived mAbs also detected bilirubin in CAV explants, with staining intensity roughly proportional to bilirubin reactivity in the ELISA platform. Collectively, these observations raise the possibility that locally produced antibodies targeting bilirubin play a pathogenic role in this disease.

## Heme catabolism in heart transplants: a double-edged sword?

**Figure 1:**
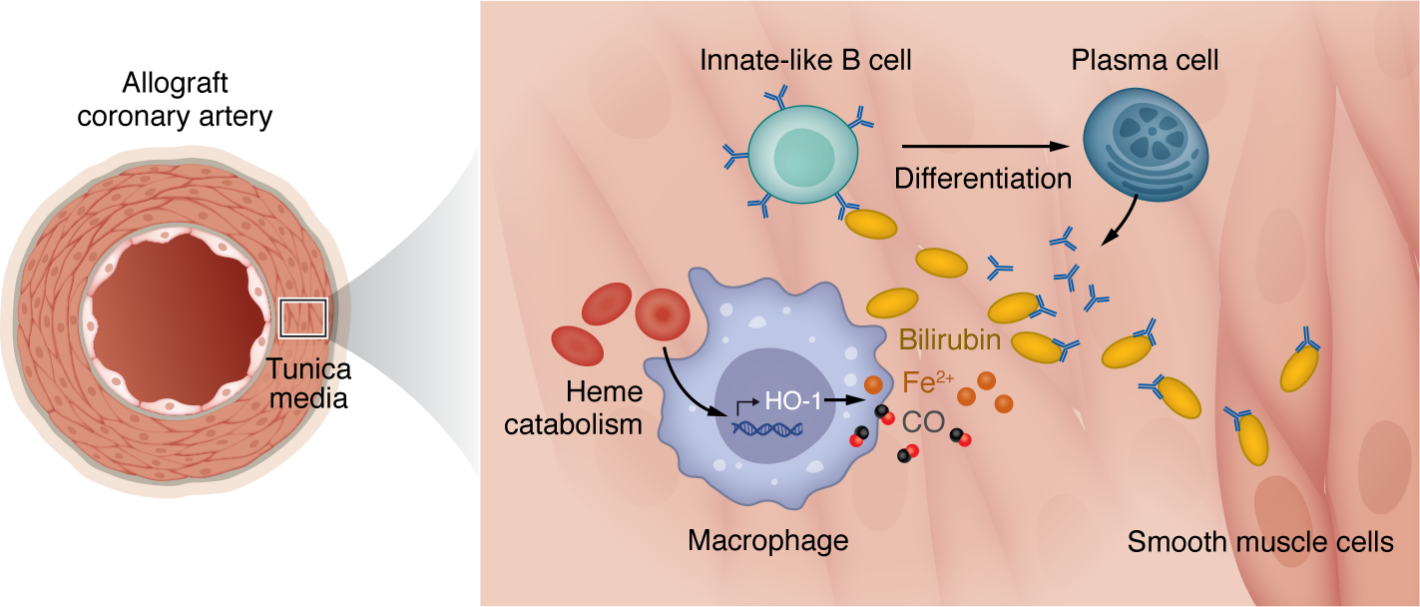
Schematic of intragraft heme catabolism and bilirubin-specific antibody production during CAV pathogenesis. See et al. demonstrated that intragraft plasma cells are derived locally from graft-infiltrating innate-like B cell clones that recognize bilirubin (6). Heme catabolism also likely occurs within allograft tissue, as evidenced by bilirubin and iron deposits in the tunica media of the coronary artery and macrophages, upregulating genes that drive heme degradation, such as HMOX1 (encoding HO-1). CO, carbon monoxide.

Remarkably, hyperbilirubinemia is a common complication after heart transplantation and cardiac failure. However, recipients with CAV did not develop hyperbilirubinemia in this study, suggesting that heme degradation is occurring in situ. This would be unexpected, as heme catabolism is commonly observed in macrophages in the spleen, liver, and bone marrow, which phagocytize dying erythrocytes and then oxidize the heme ring via heme oxygenase-1 (HO-1). Heme catabolism results in the production of a ferrous iron (Fe^2+^), carbon monoxide, and the tetrapyrrole biliverdin, which is catabolized to bilirubin by biliverdin reductases. In an examination of macrophages residing within CAV lesions, the group found compelling evidence of heme degradation as indicated by adjacent Fe^2+^ deposits, especially around infiltrating macrophages that expressed HO-1 (Figure 1). A bit more surprising was that heme catabolic pathways were also upregulated in other cells. Cytoplasmic bilirubin staining was observed in some intragraft B cells and PCs. In addition, gene expression enrichment analysis indicated a high probability of heme catabolism in intragraft-activated B cells and PCs, as evidenced by elevated transcript levels of heme oxygenase and biliverdin reductase isoforms.

The observations of intragraft heme processing raise some interesting questions about the pathogenesis of CAV. For instance, is local heme catabolism a compensatory response to allograft inflammation? Among heme catabolism’s products, bilirubin is itself a potent antioxidant, while Fe^2+^ drives the expression of the cytoprotective gene ferritin, and carbon monoxide promotes vascular relaxation to increase perfusion (8). The induction of intragraft HO-1 expression has been shown to prevent signs of cellular stress and chronic rejection in a mouse cardiac allograft model (9). Nevertheless, antibody-targeted responses to a molecule absent in the tolerant graft are likely to have deleterious consequences for allograft survival.

Clearance of heme is a normal homeostatic function of reticuloendothelial organs, indicating that antibody recognition of bilirubin may reflect a loss of self tolerance. However, it has been reported that humans have circulating natural antibodies to bilirubin (5), suggesting innate-like B cell clonal recognition of heme catabolism may have arisen from polyreactive repertoires that evolved to sense products of bacterial metabolism. Interestingly, *Corynebacterium diphtheriae* and *Pseudomonas aeruginosa* are highly dependent on scavenging iron from exogenous heme. While these pathogens do not encode the complete set of enzymes required to convert heme to bilirubin, they do express homologs of the mammalian HO-1 enzyme and produce biliverdin (10, 11). Other work has shown that *Mycobacterium tuberculosis* hijacks endogenous macrophage HO-1 activity by expressing its own functional biliverdin reductase analogue to generate bilirubin, which, in turn, is thought to promote immune evasion (12). Given that the polyreactive repertoires of innate-like B cells can exhibit broad specificities and that biliverdin and bilirubin are highly similar in structure, it would be interesting to know if intragraft-derived or natural antibodies that recognize bilirubin also bind to biliverdin.

Finally, do the products of heme catabolism mark injured cells for removal? Of note, innate-like B cells produce natural antibodies that bind to oxidized cellular membranes by recognizing the covalent adducts, such as malondialdehyde (MDA). This activity typically promotes the phagocytic removal of cellular debris to help maintain tissue homeostasis (5, 13). However, in the setting of chronic tissue injury, antibodies that recognize MDA adducts are thought to promote pathogenic organ damage in Systemic Lupus Erythematosus (14) and Coronary Artery Disease (15). Determining whether graft-derived bilirubin-specific antibodies function analogously and if bilirubin adducts are attached to cardiac-specific macromolecules should provide valuable additional insight into CAV pathogenesis.

## Conclusions

See and colleagues have identified intragraft bilirubin deposition as a major target of graft-derived antibody production in CAV (6). The investigators also provided clear evidence for the clonal relationship between intragraft innate-like B cells, locally formed PCs, and graft-derived antibody production against bilirubin. These findings are impactful and provide a basis for targeting intragraft PC development and antibody production as a therapeutic approach to combat CAV.

## Funding support

This work is the result of NIH funding, in whole or in part, and is subject to the NIH Public Access Policy. Through acceptance of this federal funding, the NIH has been given a right to make the work publicly available in PubMed Central.

- The National Institutes of Health (P01AI116501, R01HL167277, R01HL094601, R01HL173976, and P41EB025815) (to AEG).
- The Cystic Fibrosis Foundation (GELMAN25XX0) (to AEG).
- The Barnes-Jewish Foundation Maritz Chair of Immunology (to AEG).

## Version 1 02/02/2026

Electronic publication
